# Comparison of the Effectiveness of Paper Strengthening with Gelatin, Klucel G, and Tylose Solutions in Combination with Deacidification Using Magnesium Hydroxide Nanoparticles

**DOI:** 10.3390/ma19010126

**Published:** 2025-12-30

**Authors:** Renata Wojech, Aleksandra Kwiatkowska, Grzegorz Cofta, Adam Wójciak

**Affiliations:** Department of Wood Chemical Technology, Faculty of Forestry and Wood Technology, Poznań University of Life Sciences, Wojska Polskiego 38/42, 60-637 Poznań, Poland; renata.wojech@up.poznan.pl (R.W.); ola-kwiatkowska@o2.pl (A.K.); grzegorz.cofta@up.poznan.pl (G.C.)

**Keywords:** paper strengthening, deacidification, alkaline nanoparticles, cellulose, Klucel G, Tylose, gelatin

## Abstract

The manuscript concerns modern methods of preserving historical papers and presents research focusing on the effectiveness of paper strengthening with gelatin, Klucel G, and Tylose solutions in combination with deacidification using magnesium hydroxide nanoparticles. The aim of these procedures is to extend the durability of historical records on papers, which are an important part of humanity’s cultural heritage. Gelatin and Klucel G dissolved in propyl alcohol were used simultaneously with the dispersion of Mg(OH)_2_ nanoparticles, and Tylose dissolved in water was applied after deacidification in a separate step. The experiments were conducted on Whatman model papers, artificially acidified or covered with iron gall ink. The evaluation of the effectiveness was based on tests of breaking length, changes in the DP_visc_ of cellulose, and pH of the aqueous extracts. Additional information was provided by microscopic examinations (SEM-EDX-SE) and measurements of the optical properties of the tested papers before and after the application of strengthening agents. All the strengthening agents tested increased paper strength—Tylose to the greatest extent, followed by Gelatin, and Klucel G to the least extent. Model papers covered with Klucel G showed good dimensional stability. Gelatin-covered papers showed the greatest changes in optical properties.

## 1. Introduction

The combination of deacidification and strengthening of historical papers has been well-established both in practice and scientific literature pertaining to single-item paper conservation [1,2,3,4,5]. The aim of deacidification is to neutralize acids that catalyze the hydrolytic degradation of cellulose, thereby inhibiting fiber deterioration and ultimately preventing further weakening of the paper’s strength [6,7,8,9,10]. Deacidification is most commonly carried out using washing or spraying techniques, although the deacidifying agent can also be applied with a brush. The washing technique, however, weakens paper sheets. This weakening does not pertain to the paper’s strength alone but also to the inscriptions on it, which may be sensitive to the conservation techniques used. Single-item paper conservation is usually used with the most valuable artifacts in poor condition, requiring the utmost care in handling. Although individual paper conservation techniques may seem like relatively simple procedures, they require considerable expertise in conservation science. In the strengthening process (sizing/lining), Japanese tissue and adhesive should be selected in such a way as to ensure the item’s appropriate strength, dimensional stability, and minimal alterations in optical properties. The combined process of deacidification and strengthening is expected to extend the durability of historical paper for even hundreds of years.

Various deacidification methods can be used. However, when employing a spray, deacidification combined with strengthening will typically be a two-step process (Step I—deacidification, Step II—strengthening). With washing or brushing, the sequencing of treatments will depend on the mutual solubility of the deacidifying agent and the adhesive in water or other solvent. When carrying out deacidification and strengthening using nanoparticles, it is also necessary to consider the ability of the adhesive-dissolving solution to form a dispersion. For example, it would be challenging to combine deacidification using dispersions in alcoholic solutions with strengthening using a water-soluble adhesive. This study presents the results of tests on combined deacidification and strengthening treatments for paper, carried out as both one-step and two-step processes.

In terms of strengthening agents, conservators can use various modified cellulose-based solutions and glutin-based adhesives, such as gelatin. Gelatin appears to be particularly useful for the consolidation and strengthening of manuscripts written with iron gall ink. It slows down ink corrosion on paper, most likely by reducing ion mobility or forming complexes with metal ions [11,12,13,14,15,16]. Gelatin can be used as an adhesive for Japanese tissue and successful applications have been reported where a deacidifying agent was introduced alongside gelatin [14]. In addition to gelatin, modified cellulose-based agents such as methyl cellulose, ethyl cellulose, hydroxyethyl cellulose, hydroxypropyl cellulose, and carboxymethyl cellulose can be used for consolidation/strengthening and adhering Japanese tissue [1,2,3,4,5,17,18,19]. Over the past decade, numerous studies have highlighted the potential for using various new cellulose-based materials, including nanocellulose [20,21,22,23,24,25]. Research has also indicated the potential for strengthening and compensating losses in historical papers using relatively new cellulose-based preparations, such as trimethylsilyl cellulose (TMCS) in the form of a colloidal dispersion of Mg(OH)_2_ nanoparticles in hexamethyldisiloxane (HMDSO) [26]. TMCS is a controversial material due to surface hydrophobization, which can make procedures such as paper washing more difficult. However, the authors highlight its role in obtaining a stable colloid with the deacidifying agent (magnesium hydroxide nanoparticles) and improving the tensile strength of the paper. An advantage of cellulose-based agents is their chemical affinity with paper fibers, particularly with handmade papers or those produced from chemical pulp (cellulose).

The strengthening of papers with glutin-based and cellulose-based agents has been known for decades, but there is still a lack of broader comparative experimental data on the effectiveness of combining deacidification with strengthening. The combination of deacidification and consolidation/strengthening has been well established for preparations such as methyl cellulose, which is relatively water-soluble and can be used during deacidifying washing with compounds like Ca(OH)_2_ or Mg(HCO_3_)_2_. Alkaline earth metal hydroxide particles not only have the ability to neutralize acids but also slow down the oxidative degradation of cellulose [19,27,28]. This is how, e.g., magnesium compounds work, serving as free radical scavengers [29]. However, this technique cannot be used in cases where the inscription is sensitive to water. The possibilities of combining paper deacidification with strengthening or Japanese tissue lining in conservation treatment have expanded with the publication of studies and patents utilizing nanometric-sized compounds for deacidification and enhancing preservative effects [14,30,31,32,33,34,35,36,37]. It has been proposed that inscriptions sensitive to water should be treated with dispersions of alkaline earth hydroxide and oxide nanoparticles in alcohols [38,39,40,41,42,43,44,45,46,47,48]. Nanoparticle dispersions will not neutralize acids present in paper as effectively as true hydroxide solutions, but in the case of deacidifying papers with water-sensitive inscriptions, they are difficult to replace. Dispersions of nanoparticles in alcohol can also be useful when strengthening materials soluble in alcohols (e.g., Klucel or gelatin). Water-soluble strengthening agents such as methyl cellulose or hydroxyethyl methyl cellulose (e.g., Tylose) cannot be used simultaneously with nanoparticles due to the potential precipitation of hydroxide or oxide deposits in water solutions. However, deacidification can be used as a preliminary stage of paper conservation, followed by strengthening as a subsequent step.

The aim of this study is to compare the effectiveness of strengthening and deacidification treatments on model papers using preparations soluble in propyl alcohol (gelatin and Klucel G) and in water (Tylose). Gelatin and Klucel were applied simultaneously with a dispersion of Mg(OH)_2_ nanoparticles, while Tylose was applied after the deacidification process in a separate stage.

## 2. Materials and Methods

### 2.1. Model Papers

Two types of model papers were used in the study. These were subjected to acidification treatment in the laboratory of the Department of Chemical Wood Technology at the Poznań University of Life Sciences to replicate the typical pH of actual acidic papers:

- Whatman^TM^ 3MM Chr blotting paper (Merck, Darmstadt, Germany, catalog no. 3030-917), made from cotton linters containing 98% α-cellulose, with a basis weight of 180 g/m^2^ (the basis weight within the sheet area ranged between 180 and 184 g/m^2^) and a thickness of 0.35 mm, allowing for cross-sectional microscopic analysis and optical property measurements. The initial pH (cold water extract) of the paper was 7.36;
- Whatman^TM^ 2 Chr blotting paper (Merck, Darmstadt, Germany, catalog no. 3002-317), made from cotton linters containing 98% α-cellulose, with a basis weight of 100 g/m^2^ (the basis weight within the sheet area ranged between 100 and 107 g/m^2^) and a thickness of 0.19 mm, primarily used for strength testing (tensile strength) and intrinsic viscosity measurements. The initial pH (cold water extract) of the paper was 7.22. In the Whatman^TM^ model papers mentioned above, only trace amounts of magnesium (<0.0005 mass%, within the margin of measurement error) were detected using the ICP-AAS method. Furthermore, the XPS spectra did not show the presence of Mg on the surface of the acidified samples (the methodology for AAS and XPS determinations has been described elsewhere: [47,48].

**Figure 1:**
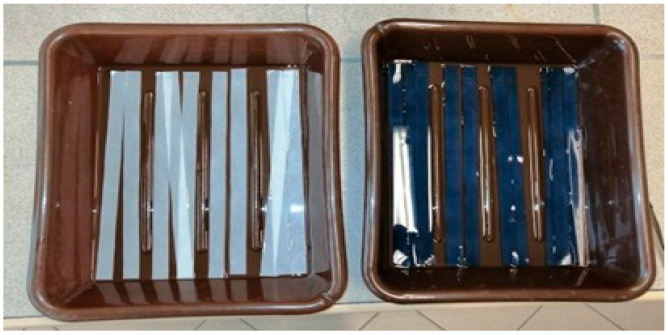
Acidification of model paper samples for breaking length measurements.

The paper samples, cut according to standard requirements for strength tests and established experimental conditions, were acidified with an H_2_SO_4_ solution (initial pH of the solution: 2.07) for 34 h until a pH level of 4.00–4.50 was achieved (cold water extract pH of the model paper samples). The acidification was carried out in photographic trays (Figure 1), with the paper samples fully immersed in the acid solution for the entire duration of the process. Some of the non-acidified samples were covered with iron gall ink prepared according to Johan G. Neevel’s method [49].

**Figure 2:**
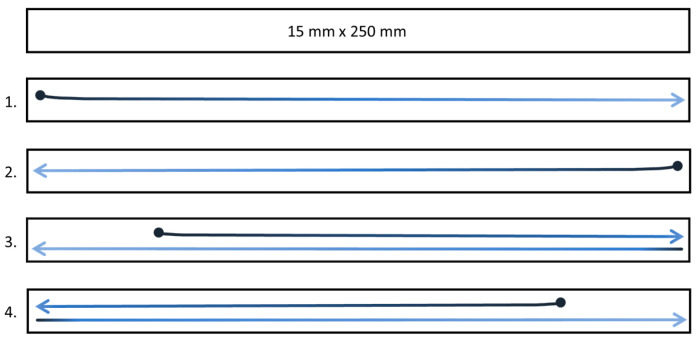
Method of covering model paper with ink for breaking length determinations.

For breaking length determination, the ink was applied to the samples using the following method (Figure 2):

- The first immersion of the brush in the ink;
- The brush was positioned on the left side of the sample’s longer edge and, with a smooth motion, the ink was applied along its length to the end of the sample (1);
- The second immersion: the application process was similar, but this time, it started from the opposite side (2);
- The third immersion: ink application began five centimeters from the beginning of the left side of the sample; with a smooth motion, the brush was moved across 15 cm, then rotated and moved to the end of the sample (3);
- The fourth immersion: the ink was applied to the samples in the same manner as after the third immersion, but this time from the opposite side (4).

**Figure 3:**
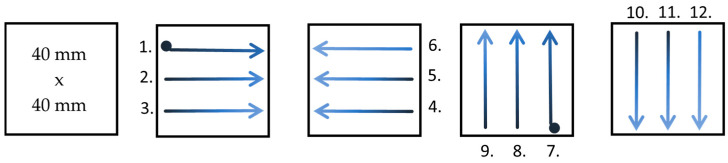
Method of covering model paper with ink for optical properties measurements.

For optical properties, pH, and moisture determination, the ink was applied to the samples in a similar way (Figure 3):

- The first immersion of the brush in the ink;
- The brush was positioned at the top left side of the sample and the ink was applied along the length of the sample to the end; this process was repeated three times, moving from left to right, each time placing the brush beneath the previous ink layer (1–3);
- At the point where the previous step ended, the brush was rotated, and the ink was applied from right to left three times, moving upward (4–6);
- The second immersion: the ink was applied from the right side of the sample, moving upward from bottom to top; the brush was positioned three times next to the preceding ink layer (7–9);
- Following the previous step, the ink was applied from top to bottom (10–12).

The ink was applied to filter paper, and the samples were conditioned for 10 days. After determining the moisture content, the paper samples were weighed before and after application to assess the amount of ink introduced. All model papers used for breaking length determination showed the same amount of ink applied, i.e., 0.0007 g/cm^2^.

Artificially acidified paper samples were left between sheets of neutral filter paper at room temperature for 10 days until fully dried. The samples were gently pressed between layers of paper sheets in cardboard folders, with a load of approximately 1.2 g/cm^2^, ensuring a uniform surface. After this period (10 days), the paper samples were deacidified and/or treated with paper-strengthening agents.

### 2.2. Deacidifier and Paper-Strengthening Agents

**Figure 4:**
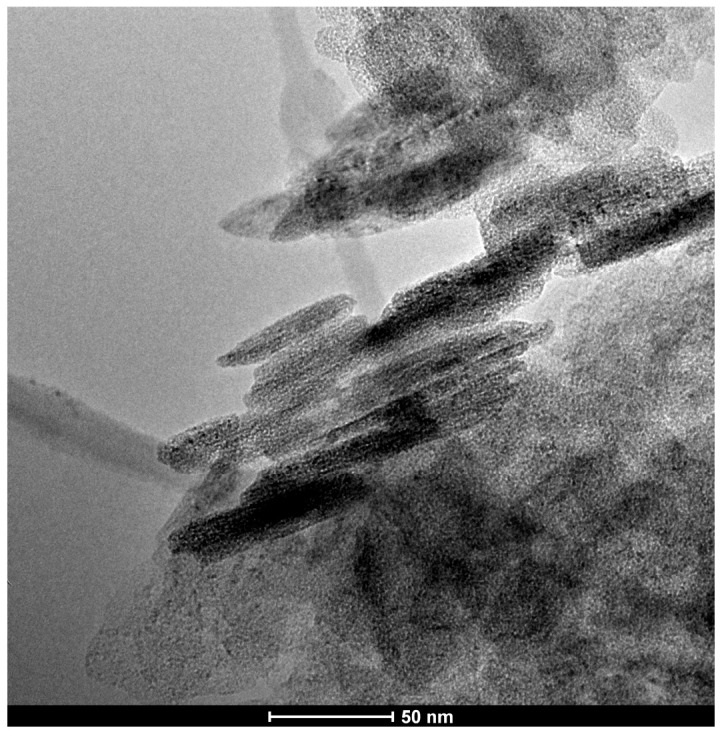
TEM image of Mg(OH)_2_ nanoparticles used for paper deacidification.

Nanoparticles of Mg(OH)_2_ (Aldrich, St. Louis, MO, USA, catalog no. 632309) with molecules below 100 nm (as measured by laser Particle Size Analysis, TEM, XRD—supplier information) were used as the deacidifier. The TEM image shows the size of the flat-shaped Mg(OH)_2_ particles (Figure 4). Qualitative and quantitative analysis using TEM with an EDX spectrometer confirmed that magnesium and oxygen are the essential components of the particles used.

Strengthening agents:

- some samples were strengthened using only a hydroalcoholic solution of gelatin obtained by the alkaline method (gelatin from bovine skin, Type B, AppliChem GmbH, Darmstadt, Germany, catalog no. A1693,0500) or a hydroalcoholic gelatin solution combined with Mg(OH)_2_ deacidifier;
- other samples were treated with either an alcoholic solution of hydroxypropyl cellulose (Klucel^®^ G; Hercules Inc., Wilmington, WA, USA) or a hydroxypropyl cellulose solution combined with Mg(OH)_2_ deacidifier;
- the study also used hydroxyethyl methyl cellulose (Tylose^®^ MH 300, [MHEC], SE Tylose GmbH & Co. KG, Wiesbaden, Germany), but Mg(OH)_2_ was introduced into the paper samples in a separate, initial stage of the experiment, followed by the strengthening treatment.

Solutions of gelatin and cellulose-based agents, both with and without the deacidifying agent, were applied to acidified or ink-covered paper samples. The following strengthening agent concentrations were used:

- 3% hydroalcoholic solution of gelatin;
- 1% solution of cellulose-based agent in 2-propanol (Klucel G) or in water (Tylose).

The concentration range of magnesium compounds (Mg(OH)_2_) introduced during deacidification combined with strengthening varied from 0.0125% to 0.5%.

The concentrations of gelatin solutions, modified cellulose-based agents, and magnesium compounds were selected based on literature data [1,4,5,14] and the authors’ own experience.

Preparation of gelatin with the deacidifying agent [14]:

- 3 g of gelatin was weighed and added to a beaker containing 60 cm^3^ of distilled water, then mixed for 1 min. The gelatin was left to rest for 5 min and then heated in a water bath at 45 °C for 20 min. Every few minutes, the contents of the beaker were stirred with a glass rod to maintain the clarity of the solution. A 0.5 g sample of Mg(OH)_2_ nanoparticles was added to a beaker containing 20 cm^3^ of 2-propanol (analytical grade), stirred with a glass rod for 5 min, and then introduced into the gelatin solution. The entire contents of the beaker (gelatin and Mg(OH)_2_) were stirred for further 5 min, with the volume adjusted using 2-propanol until a gelatin concentration of 3% was achieved. The pH of the solution was 6.93 (without Mg(OH)_2_).

Preparation of Klucel G with the deacidifying agent:

- 1 g of Klucel G (hydroxypropyl cellulose) was weighed in a weighing boat, while 0.5 g of nanoparticles was placed in a sealed container. Next, 100 cm^3^ of 2-propanol (analytical grade) was measured in a graduated cylinder and poured into the container with the weighed Mg(OH)_2_. The contents of the container were stirred for 5 min. Once a uniform dispersion of nanoparticles in 2-propanol was obtained, Klucel G was slowly added to the container while continuously stirring. The entire mixture was then stirred for an additional 5 min. The container was sealed and left for six days, with its contents being regularly stirred until a uniform consistency was obtained.

Preparation of Tylose:

- 1 g of Tylose MH 300 (hydroxyethyl methyl cellulose) was dissolved in 100 cm^3^ of distilled water and stirred for 30 min. Since Tylose does not dissolve in 2-propanol, nanoparticles of Mg(OH)_2_ were not added to this solution. The deacidification of model acidic papers was carried out in the first stage of the experiment. Following this process, the paper samples were strengthened using a 1% Tylose solution (pH 8.10). Although EDX spectra of the surfaces of Whatman paper samples covered with Tylose revealed the presence of calcium compounds in only one out of four samples tested, the alkaline reaction should be unequivocally associated with the presence of Ca compounds in this solution.

Paper strengthening with Tylose:

- Stage I: washing of samples in a dispersion of Mg(OH)_2_ in 2-propanol at concentrations ranging from 0.0125% to 0.5% (2 × 30 min), followed by conditioning for 10 days [47];
- Stage II: application of the Tylose solution with a brush, followed by conditioning for 10 days.

Brushing—sizing: the agents were applied in a similar way to ink, following the process presented in Figure 2. A flat synthetic bristle 1.5 cm wide Milan brush, series 321, was used for application. Before the actual treatment, the brush was moistened with the strengthening agent during preliminary trial on a model paper:

- 1 cm^3^ of the solution was drawn into a syringe, then 0.5 cm^3^ was transferred from the syringe into a beaker; approx. half of the solution from the beaker was taken up by the brush and applied along the longer edge of the sample, starting from the left side, with the brush extending 2–3 mm beyond the strip’s edge, ensuring more thorough coverage; with a smooth motion, the solution was applied continuously to the end of the strip; the remaining half of the solution from the beaker was applied to the sample from the opposite side, following the method described above;
- half of the remaining 0.5 cm^3^ of the solution was taken up by the brush and applied approx. 5 cm from the beginning of the left side of the paper strip; with a smooth motion, the brush was moved across 15 cm, then rotated and moved in the opposite direction, applying the solution to the end of the sample; next, the remaining half of the solution was taken up by the brush and applied from the opposite side of the sample, following the method described above.

In the case of strips used for breaking length determination (25 × 1.5 cm = 37.5 cm^2^), 1 cm^3^ of the solution was applied to each side of the strip.

The coating of 40 × 40 mm samples (intended for optical property testing) was applied in a manner similar to ink application (Figure 3). However, four brush strokes were made from each edge of the sample. Furthermore, a specific amount of the solution, i.e., 0.2 cm^3^, was applied to each side of the sample. Ten repetitions were performed for each solution, and a control sample was left untreated. The solutions were applied to the samples on an acrylic plate.

The paper samples were weighed before the application of the strengthening agent and again one week after application to monitor the amount of the preparation applied. The weights of the ink and solutions applied were calculated taking into account the moisture content of the paper samples. The weights of the solutions applied showed some variation (Table 1), which stemmed from manual brushing. Given the possible influence of varying amounts of the solutions on the strength test results, data analysis was conducted using two methods—breaking length and viscometric degree of polymerization (DP) measurement. The samples intended for strength testing were conditioned according to ISO 187:1990 [50].

### 2.3. pH Measurements

The pH analyses of cold water extracts were used to monitor both acidification and deacidification (TAPPI T 509 om-02) [51]. An Elmetron CP 401 pH meter (ELMETRON, Zabrze, Poland), accurate to ±0.01, was used. All samples were reanalyzed in terms of pH four years after deacidification/strengthening.

### 2.4. SEM-SE-BSE-EDX

The observation of the paper samples was conducted using scanning electron microscopy (SEM) with a LEO Electron Microscope 1430 VP (LEO Electron Microscopy Ltd., Zeiss, Germany). The samples were examined under a microscope in both un-sputtered and sputtered conditions, with a thin gold (Au) layer deposited in a vacuum; for the sputtered samples, a Secondary Electron (SE) detector was used (LEO Electron Microscopy Ltd., Zeiss, Germany). The magnesium distribution on the sample surface was examined using a scanning microscope equipped with a Backscattered Electron (BSE) detector (LEO Electron Microscopy Ltd., Zeiss, Germany), while its quantitative analysis on the paper surface was performed using an Energy Dispersive X-ray (EDX) spectrometer Quantax 200 (Bruker Corp., Billerica, MA, USA) with an XFlash 410 detector (Bruker Corp., Billerica, MA, USA). In the analysis of paper cross-sections, the samples were subjected to ten cycles of fiber peeling from the surface using adhesive tape. SEM-EDX analysis of the adhesive tape surface (MAG-POL packing tape) did not reveal any traces of magnesium compounds, indicating that the magnesium observed on the torn paper samples could not have originated from the tape. A magnesium concentration exceeding 0.5 [mass %] was considered sufficient for the quantitative evaluation of the SEM-EDX results.

### 2.5. Thermal Ageing—Breaking Length

Following deacidification and strengthening, portions of the paper samples, along with PET/Al/PE composite foil bags, were conditioned for one week at 20 ± 1 °C and 65% relative humidity (RH). After conditioning, the paper samples were enclosed within PET/Al/PE bags and sealed using an FKR-200.300 impulse sealer (DFPACK, Wenzhou, China) [52]. All edges were dual sealed to ensure the system’s air-tightness during the accelerated aging tests. Next, the bags were placed in an aging chamber (WAMED, Warsaw, Poland) and maintained for 5 min at a temperature of 80 ± 0.1 °C and approximately 65% humidity.

Changes in strength properties in the conditioning atmosphere (50% RH, 23 °C, according to ISO 187:1990 [50]) were monitored based on breaking length measurements according to the ISO 1924-2:2008 standard [53], with results expressed in kilometers.

### 2.6. Degree of Polymerization (DP) After Thermal Ageing

The viscosity/DP of cellulose in the model papers were assessed using the viscometric method (TAPPI T 230 om-89) [54]. Based on the average flow times of the cellulose solution (cupriethylenediamine hydroxide—cuen), the intrinsic viscosity was calculated, and the viscometric DP was determined using the Martin equation. The standard deviation for three independent determinations of intrinsic viscosity was below ±5.3 cm^3^∙g^−1^, corresponding to an DP value of 4.94. The expanded uncertainty for this parameter was ±10.6 cm^3^∙g^−1^, and for DP, it was 9.88. The results present the mean values from three independent measurements for each experimental variant. Each measurement result represents the mean of three repetitions of the flow time measurements (importantly, since all the results in the article originate from one series of tests under the same conditions, we decided to leave blank spaces for those DP measurements that could not be carried out within this series due to cellulose precipitation during one determination with papers not covered with ink and two determinations with papers covered withink).

### 2.7. Optical Properties

The paper’s optical properties (brightness) were evaluated seven days after strengthening using an L&W ELREPHO 2000 spectrometer (Lorentzen & Wettre, Stockholm, Sweden) in accordance with the ISO 2470 standard method [55], and color measurements were conducted based on the CIE L*a*b* color space. The same paper samples were examined before deacidification, after deacidification, and following thermal aging, with measurements taken at the same point on the paper surface. Five measurements were performed on three different paper samples for each type of treatment or reference, resulting in a total of 15 observations per treatment type or reference.

## 3. Results and Discussion

### 3.1. Remarks on the Preparation and Application of Strengthening Agents Combined with Mg(OH)_2_ Nanoparticles

The Mg(OH)_2_ nanoparticles introduced into Klucel G and gelatin solutions used in the experiments showed similar tendencies to form dispersions. However, there were differences in how these preparations interacted with the fibers of the model paper (room conditions: 20 °C, 55% RH):

- Gelatin–nanoparticles form dispersions within gelatin and do not precipitate. The formulation is easy to apply with a brush onto paper (more so than Tylose), providing good coverage and effectively penetrating the paper. During drying, the model paper (Whatman) tended to wrinkle and deform, as well as adhere to smooth surfaces (plexiglass) and other paper materials (Whatman blotting paper). The model papers dried very slowly and required weighting to prevent deformation.
- Klucel G—nanoparticles form dispersions and precipitate only after several minutes (without stirring). The formulation spreads easily and is readily applied with a brush, effectively penetrating the paper. The model papers (Whatman) dried quickly and, once dry, exhibited good dimensional stability (no deformation or signs of fiber shrinkage).
- Tylose—easily applied with a brush, spreads smoothly, and penetrates the paper well. However, the test samples wrinkled and bent during drying (shrinkage, deformation), and the paper took a long time to dry at room temperature and humidity.

### 3.2. Determination of Water Extract pH

The results of pH measurements demonstrated that an alkaline reaction of water extracts can be achieved when appropriate doses of Mg(OH)_2_ are used together with the strengthening agent (Table 2). For ink-covered papers, characterized by an exceptionally low reference sample pH, the concentration of the deacidifier required for neutralization was relatively high, at 0.5%. For papers artificially acidified with H_2_SO_4_, the pH of water extracts at this concentration (0.5%) was already too high, reaching approximately pH 11. When deacidifying actual acidic papers, the concentration of the deacidifier should be adjusted according to the level of paper acidity, ensuring that the pH does not exceed 8.5, especially in items written with copper-containing ink, which catalyzes oxidative degradation of cellulose [42]. It should be emphasized that four years after the strengthening and deacidification treatments, the model paper samples still exhibited an alkaline reaction in water extracts.

### 3.3. Microscopic Analyses—Distribution of Magnesium Compounds

Although microscopic images show only a small section of the sample surface, they are nevertheless a useful tool in interpreting paper deacidification results.

**Figure 5:**
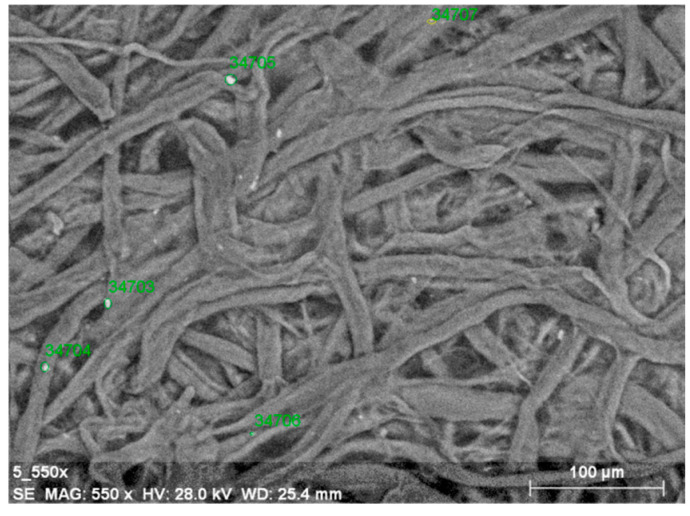
SEM-BSE image of magnesium compounds deposits on paper surface after deacidifying washing with 0.025%. Mg(OH)_2_ dispersion in 2-propanol and strengthening with Tylose (magnification ×550).

In this study, microscopic analyses indicated that the distribution of magnesium compounds, both on the surface and within the samples, is in line with the results of similar analyses performed on papers deacidified solely with a dispersion of magnesium oxide or magnesium hydroxide [47,48]. In the case of deacidification and subsequent strengthening of model papers with Tylose (Figure 5, examples of quantitative measurement points for magnesium are marked in green), the image of the sample surface reveals small magnesium compound particles, distributed sparsely but in a fairly uniform manner. The amount of magnesium is relatively small (Table 3), as the Tylose solution applied after deacidification most likely covered the agglomerates of magnesium hydroxide nanoparticles deposited during the first stage of the process (deacidifying washing). Furthermore, the image presents the sample surface deacidified at a relatively low dispersion concentration—0.025%. No magnesium compounds were observed under the surface of the sample (after ten cycles of tape peeling from the top fiber layer).

**Figure 6:**
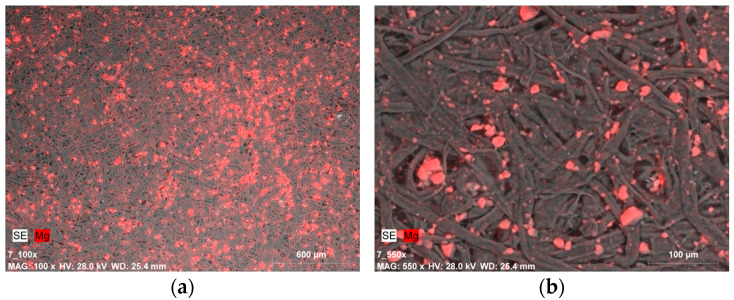
SEM-EDX image of: (**a**) magnesium compounds on the Whatman paper surface after deacidifying and strengthening with gelatin and Mg(OH)_2_ nanoparticles (0.5% Mg(OH)_2_ dispersion, magnification: ×100); (**b**) magnesium compound agglomerates (micrometric size) on the Whatman paper surface after deacidifying and strengthening with gelatin and Mg(OH)_2_ nanoparticles (0.5% Mg(OH)_2_ dispersion, magnification: ×550).

**Figure 7:**
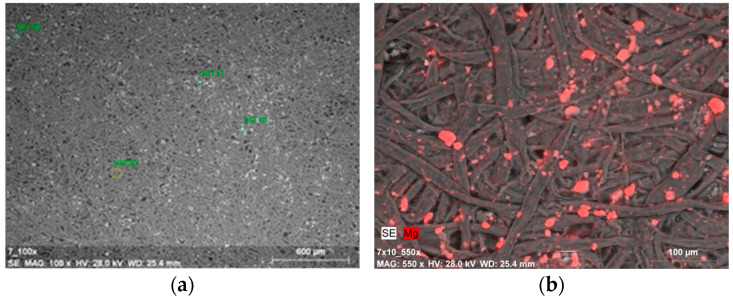
(**a**) SEM-SE image of deposits on paper surface after deacidifying and strengthening with gelatin and Mg(OH)_2_ nanoparticles (0.5% Mg(OH)_2_ dispersion, magnification: ×100); (**b**) SEM-EDX image of magnesium compound agglomerates on the Whatman paper after deacidifying and strengthening with gelatin and Mg(OH)_2_ nanoparticles (0.5% Mg(OH)_2_ dispersion, magnification: ×550)—the image shows the paper sample after peeling off fibers from the surface with the use of adhesive tape.

A completely different situation is presented in the images of samples covered with gelatin along with a 0.5% dispersion of Mg(OH)_2_ nanoparticles (Figure 6a and Figure 7a). The distribution of magnesium compounds, despite being applied with a brush, is fairly uniform. The nanoparticles appear in the form of micrometric-sized agglomerates (Figure 6b).

The amount of magnesium in point measurement areas varied within a fairly wide range, from just over 2 mass% to over 13 mass% (Figure 7a and Table 3). The local differences in the amount of measured magnesium may be related to different sizes of agglomerates, which can be seen in photographs taken at higher magnifications (×550). The image of a sample after ten cycles of tape peeling from the top fiber layer points to an effective incorporation of nanoparticles into the fibrous structure of the paper (Figure 7b). The microscopic findings were similar to those obtained for model papers deacidified solely with a dispersion of nanoparticles—without strengthening [48].

**Figure 8:**
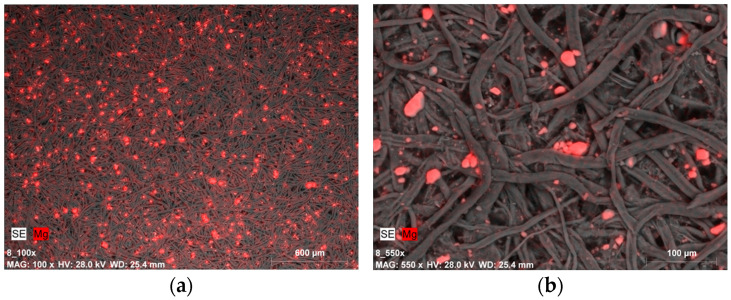
SEM-EDX image of: (**a**) magnesium compounds on the Whatman paper surface after deacidifying and strengthening with Klucel G and Mg(OH)_2_ nanoparticles (0.5% Mg(OH)_2_ dispersion, magnification: ×100); (**b**) magnesium compound agglomerates (micrometric size) on the Whatman paper surface after deacidifying and strengthening with Klucel G and Mg(OH)_2_ nanoparticles (0.5% Mg(OH)_2_ dispersion, magnification: ×550).

Similar images in terms of magnesium compound distribution and quantity were recorded for samples covered with Klucel G. Most samples exhibited a certain uniformity in magnesium distribution, especially at lower magnifications (Figure 8a), as well as the presence of magnesium compound agglomerates (Figure 8b).

**Figure 9:**
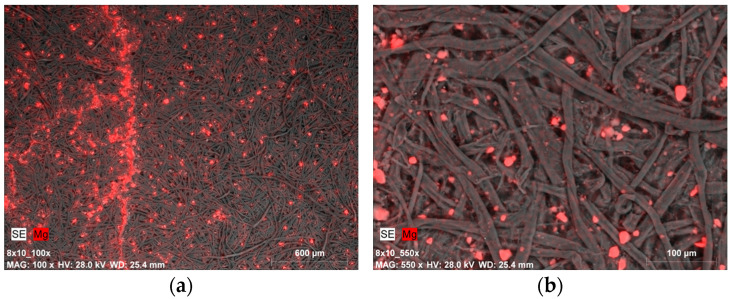
SEM-EDX image of magnesium compound agglomerates on the Whatman paper after deacidifying and strengthening with Klucel G and Mg(OH)_2_ nanoparticles (0.5% Mg(OH)_2_ dispersion): (**a**) the effect of applying the preparation with a brush, magnification: ×100; (**b**) the image shows the paper sample after peeling off fibers from the surface with the use of adhesive tape, magnification: ×550.

The image of a sample after ten cycles of tape peeling from the top fiber layer points to an equally effective incorporation of nanoparticles into the fibrous structure of the paper, similar to the use of gelatin with an Mg(OH)_2_ dispersion (Figure 9b). The size of the agglomerates is associated with the application method (brush) along with the strengthening agent—nanoparticles had to cluster and merge into larger formations, yet they still proved capable of penetrating the porous structure of the sample. In one of the images, also taken for a sample after ten cycles of tape peeling from the top fiber layer, a distinct streak with a higher concentration of magnesium compounds was observed, which is likely an effect of brushing. This indicates the difficulty in achieving uniform distribution of the solutions using a manual brushing technique (Figure 9a).

Magnesium compounds applied together with a liquid solution penetrated the paper structure more effectively than in the case of deacidification alone (without strengthening). The magnesium content determined by the EDX method on the surface and within the paper was comparable for the lowest recorded concentrations in point measurements and slightly lower for the highest concentrations determined using the same measurement method (point measurement at the site of agglomerate observation) (Table 3). Similar measurements for papers that were only deacidified revealed a clear reduction in magnesium content beneath the paper surface—magnesium compounds accumulated mainly on the surface [48].

### 3.4. Breaking Length and DP

Due to the various forces acting on paper products during use, they need to undergo a range of mechanical strength tests. The mechanical properties of paper depend on different factors (including fiber morphology, DP of cellulose, chemical composition of fibers, presence of fillers, and production technology). Breaking length refers to the critical length of a paper strip, beyond which the strip, when suspended at one end, tears under its own weight. This parameter illustrates paper’s ability to resist breaking under tension. These tests can also be used to calculate the tensile strength index, which is now a more commonly used metric. In the context of paper technology, breaking length depends on several factors, including the degree of fiber refining and the associated processes of internal and external fibrillation, the presence of fine fractions, the increase in fiber flexibility, and the formation of physico-chemical bonds between fibers (hydrogen bonds and van der Waals forces). Breaking length, similar to tensile strength, depends on the quantity and strength of fiber bonds and, to a lesser extent, on fiber length.

The results of breaking length and DP measurements indicate certain recurring dependencies, observed in both artificially acidified paper samples (Table 4) and ink-covered samples (Table 5). The introduction of cellulose-based agents (Klucel G, Tylose) and gelatin, both in water and 2-propanol environments, likely enhanced fiber bonding capacity by filling voids within the paper structure and increasing the number of physico-chemical bonds. It is evident that the formation of fiber bonds in the paper samples tested was also influenced by the functional groups present on the surface of modified cellulose-based agents and within the structure of gelatin proteins ([56], p. 70). Cellulose ethers can be regarded as polyelectrolytes, more precisely polyacids, which adsorb onto the surface of cellulose fibers due to the attraction of oppositely charged ions. However, this mechanism is more complex and should take into account the spatial conformation of polyelectrolytes on the fiber surface ([56], pp. 147–157). Another factor that contributed to the improvement of the breaking length parameter was undoubtedly deacidification, which limited the hydrolytic degradation of cellulose and the breaking of bonds within cellulose chains. This resulted in a higher DP, and consequently, greater tensile strength of individual fibers and paper. The clear trend of increased strength properties due to magnesium hydroxide nanoparticle dosing in the process of deacidification and strengthening with Tylose is illustrated by the results in Table 4 and Table 5. The comparison of different strengthening methods and agents indicates that the two-step process using Tylose had the most favorable impact on the tensile strength index. Less favorable results were obtained with gelatin, followed by Klucel G. Given that two treatments in a solvent-based environment (deacidification in 2-propanol and the application of Tylose in water) should theoretically lower the strength indicators of the model papers to a greater extent, it appears that the breaking length differences observed are related to the varying binding capacity of individual strengthening agents. However, given the manual application method, which does not ensure uniform coverage, and the potential impact of the varying solution amounts on the strength test results, it is not possible to definitively determine whether, for example, gelatin application provides greater strength compared to the Klucel G agent.

### 3.5. Impact of the Solutions on the Optical Properties of the Model Papers

The goal of conservation treatments is not so much to extend the durability of the paper itself, but primarily to preserve the inscription or image applied to it. From this perspective, it is crucial that conservation and restoration treatments should not diminish the readability of the document. In cases where a compromise is necessary, e.g., lining with Japanese tissue, the impact on legibility should be minimal.

The L* and b*, and R457C values (Table 6 and Table 7) were considered significantly different from one another when they exceeded the range of two standard deviations (the 2σ rule provides a 95% probability of assessing a significant difference). The analysis of paper samples artificially acidified up to pH 4.5 (Table 6) showed that the solutions applied significantly reduced paper brightness. Samples covered solely with gelatin darkened to the greatest extent, while those treated with gelatin with an addition of white Mg(OH)_2_ nanoparticles showed slightly less discoloration. Furthermore, samples covered with gelatin (both with and without Mg(OH)_2_) exhibited noticeable yellowing, as reflected in the increased b* value. The application of the other cellulose-derived agents (Klucel G and Tylose) had a lesser effect on the b* value. However, a slight yellowing trend was observed exclusively in the samples that did not contain naturally white Mg(OH)_2_ nanoparticles.

Similar results, but even more distinctly illustrating the trends in optical property changes, were obtained for papers covered with iron-gall ink (Table 7): a reduction in lightness (L*) and ISO brightness (R457C) was observed in all samples, along with yellowing in papers treated with gelatin (increase in the b* value). Since the tests for ink-covered samples were conducted relatively soon after the ink was applied and covered the entire surface of the model paper, these data cannot be directly correlated with changes in the legibility of the inscription. This issue requires separate aging tests, including those on samples with ink inscriptions. What is important is that an increased dose of nanoparticles with a naturally white color increased the brightness of the model papers, as demonstrated by the measurement results for two Tylose strengthening variants (0.0125% and 0.025% Mg(OH)_2_).

Although tests on the model paper samples revealed significant differences in brightness after the strengthening treatment, in most cases, these changes remain imperceptible to the average observer (Table 8). The results of color difference index (ΔE) calculations for the Whatman model papers (non-acidified at pH 7.36 and acidified up to pH 4.5) indicate that, for the human eye, only the gelatin-covered paper samples appear visibly altered. These results show the need for caution when using gelatin, especially in the case of papers with aesthetic and artistic significance. Cellulose-based agents did not alter the optical properties in a way that would be perceptible to the average observer. The interpretation of the ΔE index values differs for samples covered with ink and then strengthened. The use of the agents tested in this study on papers with ink inscriptions may alter our perception of those inscriptions. Determining how these agents affect inscriptions in such documents requires separate testing, including tests on authentic or experimental items written in ink. It should be noted that a study on changes in the optical properties of samples treated with strengthening agents and subsequently aged through exposure to temperature and light will be the subject of a separate research paper.

## 4. Conclusions

- Both gelatin and Klucel G form stable dispersions with magnesium hydroxide (Mg(OH)_2_) nanoparticles. The solutions are easy to apply with a brush onto paper (more so than Tylose), providing good coverage and effectively penetrating the paper. During drying, the model paper covered with gelatin, and to a lesser extent with Tylose, tended to wrinkle and deform, as well as adhere to smooth surfaces (plexiglass) and other paper materials (Whatman blotting paper). In the case of Klucel G, the model papers (Whatman) dried quickly and, once dry, exhibited good dimensional stability (no deformation or signs of fiber shrinkage);
- Gelatin and Klucel G, when used with an alcoholic dispersion of magnesium hydroxide (Mg(OH)_2_) nanoparticles, effectively deacidify acidic papers. The effectiveness of deacidification depends on the quantity of magnesium hydroxide nanoparticles used;
- All the strengthening agents tested caused an increase in paper strength—Tylose to the greatest extent, followed by gelatin, and Klucel G to the least extent. The quantity of Mg(OH)_2_ nanoparticles influenced the strength properties (breaking length) and DP of the model papers;
- Gelatin visibly darkens and yellows the paper and the ink inscription. These changes are perceptible to the average observer. Klucel G and Tylose also reduce paper brightness, but in a manner that is not perceptible to the average observer.

## Figures and Tables

**Table 1:** The weights of strengthening agents applied to model papers for breaking length determination (average results for 10 measurements).

Strengthening Agent	Paper Covered with Ink [g/cm^2^]	Acidified Model Papers [g/cm^2^]
Gelatin 3% + Mg(OH)_2_ 0.5%	0.0012	0.0013
Klucel G 1% + Mg(OH)_2_ 0.5%	0.0004	0.0008
Tylose 1% + Mg(OH)_2_ 0.0125%	0.0003	0.0005
Tylose 1% + Mg(OH)_2_ 0.025%	0.0002	0.0004

**Table 2:** Results of pH measurements—model papers not covered and covered with ink: 0—reference (artificially acidified or ink-covered sample), 1—3% gelatin + 0.5% Mg(OH)_2_, 2—1% Klucel G + 0.5% Mg(OH)_2_, 3—1% Tylose + 0.0125% Mg(OH)_2_, 4—1% Tylose + 0.025% Mg(OH)_2_, 5—1% Tylose + 0.5% Mg(OH)_2_.

pH of Paper Samples		
Samples No.	Artificially Acidified with H_2_SO_4_	
	pH After Month	pH After 4 Years
0	3.95	4.77
1	9.46	10.85
2	10.44	11.01
3	5.09	5.49
4	6.79	6.37
5	10.65	10.73
**Covered with Ink**		
0	2.87	4.06
1	7.55	8.21
2	7.87	6.98
3	2.82	4.15
4	2.88	4.27
5	6.71	8.73

**Table 3:** SEM-EDX analysis of magnesium content on the surface of samples and after ten cycles of tape peeling from the top fiber layer—Whatman model paper.

Mg [Mass%]		
Strengthening	Data Range for 3 Points on the Paper Sample Surface (Lowest and Highest Result)	
	Paper Surface	After Ten Cycles of Tape Peeling
Tylose + 0.025% Mg(OH)_2_	0.65–0.77	Within the error
Gelatin (3%) + 0.5% Mg(OH)_2_	2.11–13.74	2.03–7.52
Klucel G (1%) + 0.5% Mg(OH)_2_	2.07–13.11	1.80–8.74

**Table 4:** Results of breaking length and degree of polymerization (DP) measurements—model papers not covered with ink: 0—reference (artificially acidified sample), 1—3% gelatin + 0.5% Mg(OH)_2_, 2—1% Klucel G + 0.5% Mg(OH)_2_, 3—1% Tylose + 0.0125% Mg(OH)_2_, 4—1% Tylose + 0.025% Mg(OH)_2_, 5—1% Tylose + 0.5% Mg(OH)_2_.

Samples No.	Breaking Length [km]		
	Mean Value	σ	2σ
0	1.265	0.13	±0.26
1	3.109	0.25	±0.50
2	1.926	0.28	±0.56
3	2.800	0.47	±0.94
4	3.406	0.31	±0.62
5	8.462	0.70	±1.40
	**DP First Measurement**	**Second Measurement**	**Third Measurement**
0	256.23	283.32	283.32
1	404.57	443.96	431.32
2	282.60	282.60	322.06
3	365.54	379.60	392.15
4	437.38	423.01	463.06
5	555.05	555.05	-

**Table 5:** Results of breaking length and degree of polymerization (DP) measurements—model papers covered with ink: 0—reference (artificially acidified sample), 1—3% gelatin + 0.5% Mg(OH)_2_, 2—1% Klucel G + 0.5% Mg(OH)_2_, 3—1% Tylose + 0.0125% Mg(OH)_2_, 4—1% Tylose + 0.025% Mg(OH)_2_, 5—1% Tylose + 0.5% Mg(OH)_2_.

Samples No.	Breaking Length [km]		
	Mean Value	σ	2σ
0	1.509	0.05	±0.10
1	3.059	0.10	±0.20
2	2.055	0.06	±0.12
3	1.814	0.10	±0.20
4	1.897	0.07	±0.14
5	5.894	0.95	±1.90
	**DP First Measurement**	**Second Measurement**	**Third Measurement**
0	207.60	234.76	234.76
1	314.57	342.05	327.46
2	263.52	263.52	277.40
3	261.50	261.50	261.50
4	258.90	272.53	272.53
5	-	355.82	-

**Table 6:** The optical properties (CIE Lab and R457C) of model papers acidified up to pH 4.5: before and after the application of strengthening agents (σ—standard deviation). 1—reference (artificially acidified sample), 2—Klucel G (1%), 3—3% gelatin , 4—3% gelatin + 0.5% Mg(OH)_2_, 5—Klucel G + 0.5% Mg(OH)_2_, 6—1% Tylose + 0.0125% Mg(OH)_2_, 7—1% Tylose + 0.5% Mg(OH)_2_

Samples No.	Before Strengthening							
	L*	σ	a*	σ	b*	σ	R457C	σ
1	96.65	0.14	−0.42	0.020	2.78	0.073	87.84	0.067
2	96.75	0.117	−0.42	0.020	2.77	0.087	88.12	0.017
3	96.71	0.091	−0.44	0.009	2.71	0.073	88.10	0.025
4	96.79	0.086	−0.42	0.017	2.8	0.031	88.15	0.021
5	96.88	0.085	−0.43	0.006	2.75	0.028	88.45	0.088
6	96.8	0.060	−0.42	0.013	2.73	0.073	88.28	0.19
7	96.72	0.096	−0.44	0.011	2.83	0.037	87.95	0.083
**Samples No.**	**After Strengthening**							
1	96.65	0.14	−0.42	0.020	2.78	0.073	87.84	0.067
2	96.11	0.026	−0.38	0.014	2.82	0.023	86.53	0.041
3	95.61	0.023	−0.51	0.017	3.93	0.021	83.34	0.021
4	95.7	0.033	−0.73	0.026	4.51	0.036	83.43	0.030
5	96.54	0.046	−0.39	0.017	2.60	0.053	87.85	0.064
6	96.14	0.026	−0.39	0.016	2.82	0.042	86.61	0.047
7	96.32	0.018	−0.40	0.011	2.81	0.028	87.05	0.024

**Table 7:** The optical properties (CIE Lab and R457C) of model papers covered with iron gall ink: before and after the application of strengthening agents (sample numbering as in Table 6).

Samples No.	Before Strengthening							
	L*	σ	a*	σ	b*	σ	R457C	σ
1	42.6	0.065	2.15	0.057	−11.77	0.032	17.84	0.096
2	43.66	0.038	2.02	0.054	−11.74	0.060	18.72	0.053
3	43.13	0.026	2.08	0.061	−11.71	0.018	18.26	0.076
4	44.92	0.058	1.94	0.054	−11.83	0.042	19.85	0.064
5	44.50	0.039	1.96	0.029	−11.75	0.032	18.45	0.044
6	43.23	0.052	2.09	0.009	−11.74	0.043	18.36	0.058
7	43.90	0.064	2.04	0.016	−11.81	0.034	18.95	0.060
**Samples No.**	**After Strengthening**							
1	42.60	0.065	2.15	0.057	−11.77	0.032	17.84	0.096
2	40.08	0.025	2.04	0.018	−11.23	0.020	15.64	0.041
3	30.04	0.058	2.45	0.043	−7.89	0.047	8.24	0.067
4	25.63	0.032	1.77	0.039	−2.49	0.052	5.10	0.048
5	41.26	0.012	1.57	0.008	−10.82	0.021	16.36	0.016
6	38.09	0.024	1.57	0.019	−10.72	0.027	14.01	0.034
7	39.22	0.017	1.30	0.025	−11.01	0.044	14.94	0.052

**Table 8:** The color difference ΔE in the CIE L*a*b* system for the samples tested: 1—strengthened non-acidified model papers (pH 7.36), 2—artificially acidified model papers (pH 4.5), 3—papers covered with iron-gall ink.

Sample	ΔE		
	1	2	3
Gelatin	2.17	1.12	13.64
Gelatin + Mg(OH)_2_	1.95	2.05	21.43
Klucel G	0.58	0.64	3.62
Klucel G + Mg(OH)_2_	0.51	0.37	3.39
Tylose	0.54	0.67	5.27
Tylose + Mg(OH)_2_	0.05	0.4	4.81

## Data Availability

The original contributions presented in this study are included in the article. Further inquiries can be directed to the corresponding author.
